# Characteristics associated with the consumption of malted drinks among Malaysian primary school children: findings from the MyBreakfast study

**DOI:** 10.1186/s12889-015-2666-5

**Published:** 2015-12-30

**Authors:** Hamid Jan b. Jan Mohamed, S. L. Loy, Mohd Nasir Mohd Taib, Norimah A Karim, S. Y. Tan, M. Appukutty, Nurliyana Abdul Razak, F. Thielecke, S. Hopkins, M. K. Ong, C. Ning, E. S. Tee

**Affiliations:** Nutrition Programme, School of Health Sciences, Universiti Sains Malaysia, 16150 Kubang Kerian, Kelantan Malaysia; KK Research Centre, KK Women’s and Children’s Hospital, Bukit Timah Road, Singapore, 229899 Singapore; Department of Nutrition and Dietetics, Faculty of Medicine and Health Sciences, Universiti Putra Malaysia, 43400 Serdang, Selangor Darul Ehsan, Malaysia; School of Healthcare Sciences, Faculty of Health Sciences, Universiti Kebangsaan Malaysia, Jalan Raja Muda Abd Aziz, 50300 Kuala Lumpur, Malaysia; Department of Nutrition and Dietetics, School of Health Sciences, International Medical University, No.126, Jalan Jalil Perkasa 19, Bukit Jalil, 57000 Kuala Lumpur, Malaysia; Sports Science Programme, Faculty of Sports Science and Recreation, Universiti Teknologi MARA, 40450 Shah Alam, Selangor Darul Ehsan Malaysia; Cereal Partners Worldwide, Chemin du Viaduc 1. Prilly, Lausanne, 1008 Switzerland; Nestlé Research Center, Vers chez les Blanc, 1000 Lausanne, Lausanne, Switzerland; Nestlé R&D Center, 2655633, 29 Quality Road, 618802 Singapore, Singapore; Nutrition Society of Malaysia, c/o Division of Human Nutrition, Institute for Medical Research, Kuala Lumpur, Malaysia

**Keywords:** BMI, Children, Malted drinks, Micronutrients, Physical activity, Screen time

## Abstract

**Background:**

The consumption of beverages contributes to diet quality and overall nutrition. Studies on malted drinks, one of the widely consumed beverage choices among children in Asia, however, have received limited attention. This study aimed to examine the prevalence of malted drink consumption and explored associations of sociodemographic characteristics, nutrient intakes, weight status and physical activity levels with malted drink consumption among primary school children in Malaysia.

**Methods:**

Data for this analysis were from the MyBreakfast Study, a national cross-sectional study conducted from April to October 2013 throughout all regions in Malaysia. A total of 2065 primary school children aged 6 to 12 years were included in the present analysis. Data on two days 24-h dietary recall or record, anthropometry, physical activity and screen time were recorded. Associations between malted drink consumption and related factors were examined using binary logistic regression, adjusting for region, area, gender, ethnicity and household income.

**Results:**

Among children aged 6 to12 years, 73.5 % reported consuming malted drinks for at least once per week. Consumption of malted drinks was significantly associated with region (*χ*^*2*^ = 45.64, *p* < 0.001), gender (*χ*^*2*^ = 4.41, *p* = 0.036) and ethnicity (*χ*^*2*^ = 13.74, *p* = 0.008). Malted drink consumers had similar total energy intake but higher micronutrient intakes compared to non-consumers. High physical activity level (OR = 1.77, 95 % CI = 1.06, 2.99) and lower screen time during weekends (OR = 0.93, 95 % CI = 0.86, 0.99) were independently associated with malted drink consumption among 6 to 9 year-old children, but not among 10 to 12 year-old children. No association was observed between malted drink consumption and weight status.

**Conclusions:**

Malted drink consumption is prevalent among Malaysian primary school children, particularly higher among boys, indigenous children and those who lived in the East Coast region of Malaysia. Consuming malted drinks is associated with higher micronutrient intakes and higher levels of physical activity, but not with body weight status.

## Background

Beverage consumption contributes to diet quality and overall nutrition [1]. Trends in beverage consumption among children have changed over the past several decades [2]. Nationally representative data from the United States (1977 – 2001) found that nutrient-poor beverages such as soft drinks have displaced more nutritious beverages such as milk among children [2, 3]. A large body of literature has sought to examine the relationship between health status and the consumption of beverages such as milk, fruit and vegetable juice, and sugar-sweetened beverages [4, 5]. These beverages have been linked to body weight status in a positive and/or negative manner across studies and beverage types [4, 5]. However, malted drinks, one of the most widely consumed beverages among children and adolescents in Asia [6, 7] have received limited attention.

Malted drinks are malt-based foods manufactured by mixing malt with other cereal and legume flour with or without whole milk or milk powder and/or cocoa powder [8]. Malted drinks are positioned as nutritious beverages in the market [8]. They are often marketed as containing various nutrients, such as carbohydrate, protein, fats, vitamin A, B, C and E, calcium, iron, phosphorus and potassium [8]. Nonetheless, there is a lack of published literature on malted drink consumption. A study from Singapore indicated that more than 50 % of children aged 7 to 10 years consumed malted drinks during weekdays and weekends [6]. A recent study from Malaysia demonstrated that combined circuit training with chocolate malt drink supplementation had a significant effect on reducing a bone resorption marker, which imposed positive effect on bone health [9]. To our knowledge, no epidemiological study has focused on examining the associations of malted drink consumption with nutritional status and lifestyle behaviour.

To address this gap in the literature, our study provides new evidence on malted drink consumption patterns and related characteristics using a nationally representative sample of Malaysian primary school children. We estimated the prevalence of malted drink consumption and explored the associations of sociodemographic characteristics, nutrient intakes, weight status, physical activity levels and screen time with malted drink consumption. Findings from the current study are important to determine which are the sociodemographic characteristics that relate to malted drink consumption, to understand whether and by how much the consumption of malted drinks by children contributes to them meeting nutrient requirements, and whether it is associated with healthier characteristics of children in Malaysia. Epidemiological data show that children of lower socioeconomic status consume diets of lower quality and are therefore less likely to meet nutritional requirements [10]. Thus, the data provided in the current study will inform the need for and the targeting of intervention programs around the consumption of malted drinks by Malaysian children.

## Methods

### Study design and participants

The data presented here were obtained from the MyBreakfast Study. This was a cross-sectional study which was conducted from April to October 2013 by the Nutrition Society of Malaysia. Participants were selected using a multistage sampling method based on geographical location and ethnic group distribution. The sample size was calculated according to the total population of children aged 6 to 12 years in Malaysia derived from the Population and Housing Census 2010 [11]. Data collection was carried out in five regions of Malaysia, including Central, Southern, Northern, East Coast and East Malaysia. Schools were randomly selected based on a list of public primary schools in each of the states in Malaysia as of 31^st^ January 2011 [12]. Only children who were apparently healthy and with no physical disabilities or learning difficulties were eligible to participate in this study.

Permission to conduct the study was obtained from the Ministry of Education Malaysia and State Department of Education of all the states involved. Ethical approval was obtained from Universiti Kebangsaan Malaysia Research Ethics Committee (UKMREC), in accordance with the Declaration of Helsinki. Written informed consent was obtained from parents.

### Data collection

A study information sheet, consent form and a questionnaire on socio-demographic background were sent to parents. Parents were given one week to return the consent form as well as the socio-demographic questionnaire if they agreed to participate in the study. Anthropometric measurements of the children were conducted in school. Questionnaires on physical activity were self-administered with assistance to children aged 10 to 12 years in their classroom. For children aged 6 to 9 years, the questionnaires were answered by their parents and collected at school on the following day. A brief instruction about the questionnaire was sent to the parents through short messaging system (SMS). The completeness of the questionnaire was checked once collected. For children aged 10 to 12 years, questions with missing data were interviewed and completed on the spot. For children aged 6 to 9 years, all incomplete questionnaires were returned to their parents with a note on the part where they had missed along with an SMS to inform about the missing part. The questionnaires were then re-collected on the next schooling day.

### Socio-demographic background

Data on socio-demographic background including the child’s date of birth, gender, ethnicity, parents’ educational level and monthly household income were obtained from parents through a self-administered questionnaire.

### Anthropometric measurement

Height was measured using a stadiometer (SECA 217, Germany) to the nearest 0.1 cm, while weight was measured using a digital weighing scale (SECA Clara 803, Germany) to the nearest 0.1 kg. All measurements were taken twice and for each measurement, the mean value was used in the analysis. Body mass index (BMI) was computed using the formula: weight in kg/height in m^2^. BMI-for-age Z-scores (BAZ) were determined using WHO AnthroPlus Version 1.0.3 software and categorized using WHO Growth Reference 2007 [13]. The cut-off points for thinness and severe thinness were -2SD and -3SD respectively, while the cut-off points for overweight and obesity were +1SD and +2SD respectively [13].

### Dietary intake assessment

For children aged 6 to 9 years, a food record form was used to assess dietary intakes. This form was completed by parents. Parents were asked to record all foods and beverages that their children consumed on one weekday and one weekend day. An instruction sheet on the description of food and beverage consumed in household measurements was provided to assist parents in estimating serving sizes. For children aged 10 to 12 years, 24-h dietary recall was conducted in a one-to-one interview basis in school. Children were asked to recall all the foods and beverages consumed in the past 24 h. A food album and serving models were used to facilitate portion size estimation. The 24-h dietary recall was conducted for two days, namely one weekday and one weekend day (i.e. children were interviewed on Monday to obtain dietary data on Sunday). Malted drink consumers were defined as respondents who reported consumption of malted drinks on at least one of the record or recall days. Energy and nutrient intakes were analysed using NutritionistPro^™^ Software (Axxya Systems, 2008) based principally on the Nutrient Composition of Malaysian Foods [14]. The form (powder or liquid) and brand information of malted drinks were collected, and up-to-date nutrient values from food labels were used to estimate nutritional composition. Owing to unavailability of vitamin D content information from the Malaysian Food Composition database, vitamin D intake was analyzed based on the food product labels. Dietary data were then compared to the Recommended Nutrient Intakes (RNI) for Malaysia [15].

Percentage of RNI achievement was categorized as <80 %, 80–100 % and >100 %. Under- and over-reporters of energy intake were determined by the ratio of mean energy intake against basal metabolic rate (BMR). Estimates of BMR for different age groups of participants were calculated using formulae by FAO/WHO/UNU (1985) [16], Poh et al. (1999) [17] and Poh et al. (2004) [18]. Under- and over-reporters were identified by using cut-off points as used by Torun et al. (1996) [19]. For boys, an under-reporter is defined as having energy intake of less than 1.39 BMR. For girls, an under-reporter is defined as having energy intake of less than 1.30 BMR. An over-reporter is defined as having energy intake of more than 2.24 BMR for boys and more than 2.10 BMR for girls [19].

### Physical activity assessment

Physical activity levels in the past seven days were assessed by using the Physical Activity Questionnaire for Children (PAQ-C) [20]. The PAQ-C is a self-administered seven-day recall questionnaire designed to assess moderate to vigorous physical activity in school-aged children. For children aged 6 to 9 years, the PAQ-C were completed by parents. For children aged 10 to 12 years, the PAQ-C were self-administered at school. Total scores obtained from PAQ-C were categorized into low (1.00–2.33), moderate (2.34–3.66) and high (3.67–5.00) physical activity levels. Questions on media screen time (total number of hours per day of watching television and using computer) during weekdays and weekends were asked at the end of the questionnaires.

### Statistical analysis

Data were analyzed using SPSS software version 20 (SPSS Inc. Chicago, Illinois, US). Categorical variables were presented as numbers and percentages. Continuous variables with normal distributions were presented as means (*m*) and standard deviations (SD), whereas variables which are not normally distributed were presented as medians (*M*) and percentiles (P_5_ and P_95_). Pearson chi-square tests were used to examine the associations between categorical variables and malted drink consumption status. Differences in the amount of malted drink consumption across variables were compared using Kruskal-Wallis test. Differences in total energy and nutrient intakes between malted drink consumers and non-consumers were compared using Mann–Whitney test. Associations between malted drink consumption and related factors were examined using binary logistic regression, adjusting for confounders such as region, area, gender, ethnicity and household income. Two sided *p* < 0.05 was considered statistically significant at the 95 % confidence interval.

## Results

A total of 9592 children were invited to participate in the study and 5785 agreed to take part, resulting in a response rate of 60.3 %. Of the 5785 children who agreed to take part, we only included those who had completed data for two days food record (*n* = 2998) or 24-h dietary recall (*n* = 2334) in this study. Out of 5332 children with completed dietary data, only 38.7 % of them were found to have acceptable reported dietary data, resulting in a final sample of 2065 children.

Table 1 shows the distribution of malted drink consumption status by participant characteristics. Overall, it was found that 73.5 % of children aged 6 to 12 years consumed malted drinks at least once per week. Percentage of malted drink consumption in both age groups were similar (74.1 % in children aged 6 to 9 years; 72.7 % in children aged 10 to 12 years). Univariate analysis revealed that malted drink consumption was significantly associated with regions (*χ*^*2*^ = 45.64 *p* < 0.001), gender (*χ*^*2*^ = 4.41, *p* = 0.036), ethnicity (*χ*^*2*^ = 13.74, *p* = 0.008) and physical activity levels (*χ*^2^ = 6.92, *p* = 0.031). Screen time during weekends was found to be higher among malted drink non-consumers than consumers (*m* = 4.44 vs. 4.11 h, *p* = 0.020). No significant differences in malted drink consumption were shown across area, household income and weight status.

**Table 1:** Association between malted drink consumption (g/day) and related characteristics among Malaysian primary school children

	6–9 years old				10–12 years old				Total			
Variables	Consumers	Non-Consumers			Consumers	Non-Consumers			Consumers	Non-Consumers		
	*n* (%)	*n* (%)	*χ* ^*2*^	*p*-value^a^	*n* (%)	*n* (%)	*χ* ^*2*^	*p*-value^a^	*n* (%)	*n* (%)	*χ* ^*2*^	*p*-value^a^
Overall	899 (74.1)	315 (25.9)			619 (72.7)	232 (27.3)			1518 (73.5)	547 (26.5)		
Regions			38.54	<0.001			11.26	0.024			45.64	<0.001
Central	142 (67.6)	68 (32.4)			112 (71.3)	45 (28.7)			254 (69.2)	113 (30.8)		
Southern	174 (81.3)	40 (18.7)			113 (77.4)	33 (22.6)			287 (79.7)	73 (20.3)		
Northern	222 (65.1)	119 (34.9)			144 (64.9)	78 (35.1)			366 (65.0)	197 (35.0)		
East Coast	198 (84.6)	36 (15.4)			119 (76.8)	36 (23.2)			317 (81.5)	72 (18.5)		
East Malaysia	163 (75.8)	52 (24.2)			131 (76.6)	40 (23.4)			294 (76.2)	92 (23.8)		
Area			0.05	0.821			0.14	0.707			0.01	0.928
Urban	616 (73.9)	218 (26.1)			414 (73.1)	152 (26.9)			1030 (73.6)	370 (26.4)		
Rural	283 (74.5)	97 (25.5)			205 (71.9)	80 (28.1)			488 (73.4)	177 (26.6)		
Gender			5.58	0.018			0.21	0.643			4.41	0.036
Boys	391 (77.6)	113 (22.4)			259 (73.6)	93 (26.4)			650 (75.9)	206 (24.1)		
Girls	508 (71.5)	202 (28.5)			360 (72.1)	139 (27.9)			868 (71.8)	341 (28.2)		
Ethnic groups			19.21	0.001			5.21	0.267			13.74	0.008
Malay	640 (75.2)	211 (24.8)			393 (70.8)	162 (29.2)			1033 (73.5)	373 (26.5)		
Chinese	96 (63.6)	55 (36.4)			88 (76.5)	27 (23.5)			184 (69.2)	82 (30.8)		
Indian	58 (76.3)	18 (23.7)			46 (79.3)	12 (20.7)			104 (77.6)	30 (22.4)		
Indigenous people (East Malaysia)	103 (79.8)	26 (20.2)			87 (76.3)	27 (23.7)			190 (78.2)	53 (21.8)		
Others	2 (28.6)	5 (71.4)			5 (55.6)	4 (44.4)			7 (43.8)	9 (56.2)		
Income groups (RM)			5.95	0.203			4.66	0.324			5.32	0.256
< 1500	305 (75.9)	97 (24.1)			206 (74.4)	71 (25.6)			511 (75.3)	168 (24.7)		
1501–3500	214 (71.6)	85 (28.4)			188 (76.7)	57 (23.3)			402 (73.9)	142 (26.1)		
3501–5500	158 (74.5)	54 (25.5)			104 (71.2)	42 (28.8)			262 (73.2)	96 (26.8)		
5501–7500	105 (80.2)	26 (19.8)			50 (69.4)	22 (30.6)			155 (76.4)	48 (23.6)		
> 7500	99 (69.2)	44 (30.8)			57 (66.3)	29 (33.7)			156 (68.1)	73 (31.9)		
BMI-for-age			3.12	0.538			3.62	0.460			4.86	0.302
Severe thinness	11 (68.8)	5 (31.2)			17 (63.0)	10 (37.0)			28 (65.1)	15 (34.9)		
Thinness	56 (74.7)	19 (25.3)			52 (67.5)	25 (32.5)			108 (71.1)	44 (28.9)		
Normal	690 (73.7)	246 (26.3)			455 (72.9)	169 (27.1)			1145 (73.4)	415 (26.6)		
Overweight	72 (71.3)	29 (28.7)			61 (77.2)	18 (22.8)			133 (73.9)	47 (26.1)		
Obese	70 (81.4)	16 (18.6)			34 (77.3)	10 (22.7)			104 (80.0)	26 (20.0)		
Physical activity level			10.83	0.004			0.36	0.836			6.92	0.031
Low	185 (67.3)	90 (32.7)			186 (73.2)	68 (26.8)			371 (70.1)	158 (29.9)		
Moderate	594 (75.1)	197 (24.9)			383 (72.1)	148 (27.9)			977 (73.9)	345 (26.1)		
High	120 (81.1)	28 (18.9)			49 (75.4)	16 (24.6)			169 (79.3)	44 (20.7)		
Screen time, hours^b^												
Weekdays	4.62 (2.75)	2.27 (1.54)	0.33	0.742	4.18 (3.31)	2.33 (1.77)	0.86	0.392	2.23 (1.61)	2.30 (1.64)	0.85	0.397
Weekends	4.40 (2.38)	2.24 (1.44)	1.30	0.196	3.70 (3.08)	2.21 (1.83)	0.29	0.045	4.11 (2.71)	4.44 (3.01)	2.33	0.020

^a^based on Pearson Chi-Square test or independent *t*-test

^b^presented as mean (standard deviation)

Table 2 shows the distribution of median daily intake (g/day) of malted drinks according to the various socio-demographic, behavioural and weight status characteristics. The highest median daily intake of malted drinks was found among children from the East Coast region of Malaysia (*M* = 30.0 g) and this was significantly higher than among children from the other regions in Malaysia. Among children aged 6 to 9 years, the median intake of malted drinks in the rural area was significantly higher than the urban area (*M* = 22.4 g vs. 19.1 g respectively, *p* < 0.001). Also, 6 to 9 year-old children who were overweight had the highest median intake of malted drinks (*p* = 0.019). No significant differences in the median intake of malted drinks were observed across gender, ethnic groups, household income and physical activity levels. A larger amount of malted drinks were consumed during weekends compared to weekdays.

**Table 2:** Distribution of malted drink consumption by socio-demographic characteristics and various health-related factors among Malaysian primary school children

	6–9 years old		10–12 years old		Total	
	Malted drinks, g/d (*n* = 899)		Malted drinks, g/d (*n* = 619)		Malted drinks, g/d (*n* = 1518)	
	Median (P_5_, P_95_)	*p*-value^a^	Median (P_5_, P_95_)	*p*-value^a^	Median (P_5_, P_95_)	*p*-value^a^
Total	20.0 (6.4, 60.0)		22.4 (9.5, 60.0)		20.0 (6.4, 60.0)	
Regions		<0.001		<0.001		<0.001
Central	15.9 (6.3, 45.5)		15.0 (6.3, 45.0)		15.0 (6.3, 45.0)	
Southern	19.3 (6.5, 50.7)		30.0 (10.0, 77.1)		20.5 (6.6, 60.0)	
Northern	15.8 (6.3, 47.9)		16.8 (6.3, 58.6)		16.0 (6.3, 53.8)	
East Coast	30.0 (10.0, 67.3)		30.0 (10.0, 63.8)		30.0 (10.0, 67.3)	
East Malaysia	20.0 (9.9, 65.5)		25.9 (10.0, 64.9)		25.0 (10.0, 65.0)	
Area		<0.001		0.802		0.003
Urban	19.1 (6.3, 51.3)		22.4 (6.4, 60.0)		20.0 (6.4, 60.0)	
Rural	22.4 (6.5, 67.3)		22.5 (10.0, 60.0)		22.4 (7.5, 60.0)	
Gender		0.282		0.839		0.355
Boys	20.0 (6.4, 60.0)		22.4 (6.5, 63.5)		20.0 (6.4, 60.0)	
Girls	20.0 (6.3, 60.0)		22.4 (9.5, 60.0)		20.0 (6.4, 60.0)	
Ethnic groups		0.051		0.724		0.063
Malay	20.0 (6.3, 60.0)		21.5 (9.5, 60.0)		20.0 (6.4, 60.0)	
Chinese	15.0 (6.3, 45.0)		22.0 (6.3, 60.0)		18.2 (6.3, 47.9)	
Indian	20.0 (6.4, 64.1)		28.5 (4.8, 76.8)		23.2 (6.4, 66.4)	
Indigenous people (East Malaysia)	20.0 (7.5, 67.6)		22.4 (10.0, 65.0)		20.0 (9.9, 65.4)	
Others	37.5 (15.0, 60.0)		15.0 (10.0, 72.4)		15.0 (10.0, 60.0)	
Income groups (RM)		0.286		0.446		0.100
< 1500	20.0 (6.4, 66.5)		22.7 (7.2, 66.3)		20.0 (6.5, 65.9)	
1501–3500	22.1 (6.3, 60.0)		22.4 (8.6, 60.0)		22.3 (6.4, 60.0)	
3501–5500	19.0 (6.3, 45.1)		20.0 (6.4, 55.8)		19.0 (6.4, 45.0)	
5501–7500	20.0 (6.3, 50.9)		15.0 (9.5, 59.1)		19.5 (6.3, 52.3)	
> 7500	21.3 (6.4, 60.0)		30.0 (9.5, 72.7)		23.0 (7.8, 60.5)	
Days of week		NA		NA		NA
Weekday	22.5 (6.4, 60.0)		28.0 (9.5, 64.0)		25.0 (7.5, 60.0)	
Weekend	25.0 (6.7, 60.0)		30.0 (9.5, 66.0)		26.9 (9.5, 62.3)	
BMI-for-age		0.019		0.794		0.105
Severe thinness	15.0 (8.0, 50.0)		26.9 (6.4, 67.3)		19.5 (7.1, 64.0)	
Thinness	17.5 (6.1, 61.1)		25.9 (9.5, 64.4)		20.0 (6.3, 60.0)	
Normal	20.0 (6.4, 59.3)		20.4 (9.5, 60.0)		20.0 (6.4, 60.0)	
Overweight	27.8 (6.5, 76.5)		25.0 (6.0, 84.7)		26.9 (6.4, 82.9)	
Obese	22.2 (6.4, 51.2)		29.5 (6.5, 69.4)		22.3 (6.5, 54.1)	
Physical activity level		0.668		0.855		0.825
Low	20.0 (6.3, 60.0)		22.4 (7.7, 60.0)		22.2 (6.4, 60.0)	
Moderate	20.0 (6.4, 52.4)		22.4 (9.5, 60.0)		20.0 (6.4, 60.0)	
High	20.0 (6.3, 67.3)		20.0 (6.3, 60.0)		20.0 (6.3, 66.1)	

*NA* not applicable

^a^based on Kruskal Wallis test

Table 3 presents the comparison of median daily energy and nutrient intakes between malted drink consumers and non-consumers among different age groups. Malted drink consumers were found to have similar daily intakes of energy, protein, fat, sodium and vitamin D, but significantly higher intakes of vitamin C, thiamin, riboflavin, niacin, calcium, iron and phosphorus compared to non-consumers in both age groups. A larger daily carbohydrate intake was observed in malted drink consumers compared to non-consumers among children aged 10 to 12 years (*M* = 227.21 g vs. 216.21 g respectively, *p* = 0.023). Larger proportions of malted drink consumers were found to meet the RNI for total energy intake, protein, vitamin A, vitamin C, thiamin, riboflavin, niacin, calcium and iron as compared to non-consumers. More than 90 % of children from both groups did not meet the RNI for vitamin D (Table 4).

**Table 3:** Comparison of daily energy and nutrient intakes between malted drink consumers and non-consumers

Nutrients	6–9 years old			10–12 years old		
	Malted drink consumers (*n* = 899)	Malted drink non-consumers (*n* = 315)		Malted drink consumers (*n* = 619)	Malted drink non-consumers (*n* = 232)	
	Median (P_5_, P_95_)	Median (P_5_, P_95_)	*p*-value^a^	Median (P_5_, P_95_)	Median (P_5_, P_95_)	*p*-value^a^
Energy, kcal	1717 (1341, 2305)	1685 (1324, 2311)	0.303	1842 (1439, 2481)	1804 (1399, 2534)	0.145
Protein, g	76.21 (48.44, 140.43)	74.06 (48.55, 123.12)	0.114	78.13 (51.20, 159.01)	77.76 (46.15, 147.06)	0.457
Carbohydrate, g	210.86 (148.84, 294.28)	206.11 (137.49, 298.00)	0.192	227.21 (153.79, 318.42)	216.21 (152.96, 315.16)	0.023
Fat, g	66.35 (41.96, 96.56)	64.95 (41.68, 101.23)	0.866	68.8 (45.41, 105.48)	70.14 (44.88, 106.76)	0.850
Vitamin A, mg RE	978.12 (361.47, 1889.26)	866.68 (339.83, 1894.77)	0.002	1024.53 (381.12, 1977.13)	1032.63 (318.73, 2187.71)	0.832
Vitamin C, mg	50.74 (15.65, 169.58)	34.72 (4.67, 136.26)	<0.001	50.43 (13.15, 155.90)	33.99 (3.43, 150.52)	<0.001
Thiamin, mg	0.91 (0.51, 1.69)	0.73 (0.33, 1.80)	<0.001	0.92 (0.50, 1.67)	0.68 (0.35, 1.70)	<0.001
Riboflavin, mg	1.30 (0.77, 2.44)	1.17 (0.56, 2.92)	<0.001	1.29 (0.75, 2.49)	1.09 (0.58, 2.29)	<0.001
Niacin, mg	13.46 (7.46, 22.79)	11.32 (6.05, 23.26)	<0.001	14.10 (7.91, 25.88)	12.54 (6.80, 27.28)	<0.001
Sodium, mg	1824.83 (875.06, 3335.37)	1881.77 (930.79, 3666.17)	0.292	2030.10 (1011.56, 3809.62)	2104.22 (1057.52, 3619.97)	0.822
Calcium, mg	606.67 (329.92, 1088.71)	485.06 (237.27, 1152.42)	<0.001	591.13 (312.85, 1091.59)	496.45 (212.89, 980.32)	<0.001
Iron, mg	17.58 (9.97, 33.21)	15.70 (8.24, 32.53)	<0.001	20.42 (10.29, 38.31)	18.96 (8.73, 37.18)	0.047
Phosphorus, mg	1230.50 (638.37, 2057.25)	1093.03 (495.73, 1916.28)	<0.001	1242.43 (640.27, 2063.06)	1161.45 (482.4, 2162.81)	0.026
Vitamin D, μg	0.16 (0, 4.31)	0.08 (0, 3.96)	0.785	0 (0, 3.91)	0 (0, 5.77)	0.979

^a^ based on Mann–Whitney test

**Table 4:** Distribution of RNI achievements among malted drink consumers and non-consumers according to gender

	Boys					Girls				
Variables	RNI	Malted drink consumers		Malted drink non-consumers		RNI	Malted drink consumers		Malted drink non-consumers	
		RNI achievement, *n* (%)		RNI achievement, *n* (%)			RNI achievement, *n* (%)		RNI achievement, *n* (%)	
		<80	80–100	<80	80–100		<80	80–100	<80	80–100
Energy, Kcal										
6–9 years old	1780	19 (4.9)	372 (95.1)	6 (5.3)	107 (94.7)	1590	16 (3.1)	492 (96.9)	6 (3.0)	196 (97.0)
10–12 years old	2180	70 (27.0)	189 (73.0)	20 (21.5)	73 (78.5)	1990	89 (24.7)	271 (75.3)	2 (1.4)	137 (98.6)
Protein, g										
6–9 years old	32	0	391 (100.0)	0	113 (100.0)	32	0	508 (100.0)	0	202 (100.0)
10–12 years old	45	0	259 (100.0)	1 (1.1)	93 (100.0)	46	1 (0.3)	359 (99.7)	0	139 (100.0)
Vitamin A, mg RE										
6–9 years old	500	21 (5.4)	370 (94.6)	9 (8.0)	104 (92.0)	500	31 (6.1)	477 (93.9)	14 (6.9)	188 (93.1)
10–12 years old	600	27 (10.4)	232 (89.6)	7 (7.5)	86 (92.5)	600	45 (12.5)	315 (87.5)	10 (7.2)	129 (92.8)
Vitamin C, mg										
6–9 years old	35	83 (21.2)	308 (78.8)	45 (39.8)	68 (60.2)	35	98 (19.3)	410 (80.7)	86 (42.6)	116 (57.4)
10–12 years old	65	135 (52.1)	124 (47.9)	59 (63.4)	56 (60.2)	65	189 (52.5)	171 (47.5)	60 (43.2)	79 (56.8)
Thiamin, mg										
6–9 years old	0.9	91 (23.3)	300 (76.7)	57 (50.4)	56 (49.6)	0.9	148 (29.1)	360 (70.9)	97 (48.0)	105 (52.0)
10–12 years old	1.2	132 (51.0)	127 (49.0)	46 (49.5)	47 (50.5)	1.1	169 (46.9)	191 (53.1)	82 (59.0)	57 (41.0)
Riboflavin, mg										
6–9 years old	0.9	9 (2.3)	382 (97.7)	10 (8.8)	103 (91.2)	0.9	22 (4.3)	486 (95.7)	24 (11.9)	178 (88.1)
10–12 years old	1.3	64 (24.7)	195 (75.3)	13 (14.0)	80 (86.0)	1.0	37 (10.3)	323 (89.7)	20 (14.4)	119 (85.6)
Niacin, mg										
6–9 years old	12	51 (13.0)	340 (87.0)	33 (29.2)	80 (70.8)	12	81 (15.9)	427 (84.1)	62 (30.7)	140 (69.3)
10–12 years old	16	81 (31.3)	178 (68.7)	15 (16.1)	78 (83.9)	16	144 (40.0)	216 (60.0)	47 (33.8)	92 (66.2)
Calcium, mg										
6–9 years old	700	150 (38.4)	241 (61.6)	63 (55.8)	50 (44.2)	700	229 (45.1)	279 (54.9)	126 (62.4)	76 (37.6)
10–12 years old	1000	195 (75.3)	64 (24.7)	51 (54.8)	42 (45.2)	1000	300 (83.3)	60 (16.7)	95 (68.3)	44 (31.7)
Iron, mg										
6–9 years old	9 or 6	1 (0.3)	390 (99.7)	2 (1.8)	111 (98.2)	9 or 6	1 (0.2)	507 (99.8)	1 (0.5)	201 (99.5)
10–12 years old	15 or 10	4 (1.5)	255 (98.5)	0	93 (100.0)	14 or 9	2 (0.6)	358 (99.4)	0	139 (100.0)
Vitamin D, μg										
6–9 years old	5	372 (95.1)	19 (4.9)	108 (95.6)	5 (4.4)	5	474 (93.3)	34 (6.7)	193 (95.5)	9 (4.5)
10–12 years old	5	245 (94.6)	14 (5.4)	88 (94.6)	5 (5.4)	5	344 (95.6)	16 (4.4)	128 (92.1)	11 (7.9)

*RNI* Recommended Nutrient Intake

Table 5 shows the association of BAZ, physical activity level and screen time with malted drink consumption. There were no significant associations between BAZ and malted drink consumption in either age group. Physical activity level and screen time during weekends were independently associated with malted drink consumption among 6 to 9 year-old children. Children who participated in high physical activity levels had significantly higher odds for consuming malted drinks than those who were involved in low physical activity levels (OR = 1.77, 95 % CI = 1.06, 2.99). Children who spent more time on watching television or using computer had significantly lower odds for consuming malted drinks (OR = 0.93, 95 % CI = 0.87, 1.00).

**Table 5:** Association of body weight status, physical activity level and screen time with malted drink consumption^a^

Variables	6–9 years old		10–12 years old	
	Odd Ratio (95 % CI)	*p*-value	Odd Ratio (95 % CI)	*p*-value
BMI-for-age				
BMI-for-age Z-score	1.02 (0.92, 1.13)	0.729	1.05 (0.95, 1.18)	0.341
Physical activity level				
Low	1.00 (Reference)		1.00 (Reference)	
Moderate	1.33 (0.97, 1.82)	0.079	0.93 (0.65, 1.34)	0.711
High	1.77 (1.06, 2.99)	0.031	1.12 (0.56, 2.25)	0.757
Screen time, hours				
Weekdays	1.06 (0.94, 1.20)	0.361	1.02 (0.92, 1.14)	0.697
Weekends	0.93 (0.86, 0.99)	0.032	0.95 (0.90, 1.01)	0.133

^a^Adjusted for region, area, gender, ethnicity and household income, using multivariate logistic regression. Level of significance was set at 0.05 (two-tailed)

## Discussion

The present study is one of the first to examine malted drink consumption and associated factors among Malaysian primary school children. Nationally, 73.5 % of children reported consuming malted drinks at least once a week. Boys, indigenous children, and those who lived in the East Coast region of the country had a higher prevalence of malted drink consumption. In addition, malted drink consumers were found to have similar total daily energy intake but higher daily micronutrient intakes compared to non-consumers. High physical activity level and less screen time were associated with greater likelihood of malted drink consumption after adjustment for region, area, gender, ethnicity and household income. No association was observed between malted drink consumption and weight status among children.

There was a differential effect of gender on malted drink consumption among the younger age group of children only. This might reflect gender differences in food preferences [21] and parental influences on food choices in the early childhood stage [22]. Higher prevalence of malted drink consumption among indigenous people could be attributed to the implementation of the Malaysian Food Basket programme [23]. Malted drinks are included as one of the food items in this programme and are received by a majority of the indigenous people due to food insecurity. From this aspect, malted drinks could be perceived as healthy by indigenous parents and therefore, encouraged amongst their children. A study from the United States showed that consumption of beverages such as sports and energy drinks were more prevalent in higher income populations [24]. Another study from Canada reported that consumption of soft drinks and fruit juices were more prevalent in lower socio-economic status population as determined based on education, household income and employment status [25]. However, our results did not show any association between malted drink consumption and household income. This finding may be potentially explained by the acceptable price range of malted drinks in the market.

Our study revealed that total daily energy intake was comparable between malted drink consumers and non-consumers, and no association was found between malted drink consumption and weight status. These findings suggest that malted drink consumption did not contribute to positive energy balance and weight gain during childhood. This is in contrast to the findings from other studies which examined the association between sugar-sweetened beverages and body weight. A review article from the United States concluded that consumption of sugar-sweetened beverages promoted excess energy intake and reducing the intake of these beverages had beneficial effects on body weight [26]. On the other hand, a study from Australia reported that milk consumption among children was not associated with adverse effects on body weight measures [27]. The possible explanation can be the differential effects on satiety due to nutritional and sensorial differences between beverages [28]. Our findings support the literature around the lack of satiety of more liquid beverages such as soft drinks and greater satiety of more viscous beverages such as milk to protect against overweight [28]. It seems likely that malted drinks may be considered as more viscous beverages which may have high satiation and fullness effects, hence do not contribute to overconsumption, excess energy intake then to weight gain [29].

Children who consumed malted drinks generally had greater micronutrient intakes than non-consumers. Furthermore, malted drink consumers were more likely to meet the RNI for total energy intake, protein, vitamin A, vitamin C, thiamin, riboflavin, niacin, calcium and iron. These findings suggest that malted drink consumption can be an effective and simple dietary strategy to improve overall diet quality. However, we recognize that there is a possibility of other food choices that might drive the association with greater micronutrient intakes among the malted drink consumers, and this warrants further investigation. In the current market, most malted drinks are fortified with multiple micronutrients [8] and as such, they are commonly used as multivitamin supplementation products in several intervention studies [9, 30]. An improvement in micronutrient status of thiamin, riboflavin, niacin, vitamin C and iron were reported among 7 to 10 year-old Indian children after supplementation with fortified chocolate malt drinks [30], which is consistent with our findings. However, low vitamin D intake was prevalent in all children, suggesting that malted drinks and most other foods consumed by the children are poor sources of vitamin D. As reported by Khor et al. (2011) [31], only a few foods in Malaysia have been fortified with vitamin D. This explains the low intake of vitamin D among children which is supported by high prevalence of serum vitamin D deficiency in previous studies [31, 32]. It is possible that malted drinks could be fortified with vitamin D and used as one of the medium to improve children’s vitamin D status.

It has been reported that sedentary behaviour and extended periods of media use are associated with less healthy food preferences [25, 33]. However, our study found that high physical activity level and less screen time were associated with greater likelihood of malted drink consumption. These associations were only shown to be statistically significant among children aged 6 to 9 years, revealing the importance of life stage factors. An intervention study demonstrated that fortified chocolate malt drink supplementation improved physical performance measures in Indian children aged 7 to 10 years [30]. Our study indicated that malted drink consumption was linked with physically active behaviour in younger children, suggesting this group of children or parents may perceive malted drinks as healthful beverages. The parental and children’s perceptions on the health benefits of malted drinks may be further influenced by the malted drink advertisements which show pictures of athletes on the packaging and in mass media in Malaysia.

There are several limitations of this study that should be acknowledged. Many participants were excluded due to incomplete dietary information and misreporting of dietary data, and therefore generalizability of the findings may be limited. Only two days of 24-h dietary recalls or records were collected and this may not reflect habitual consumption patterns. Fluctuation of food intakes from day to day may lead to misclassification of participants according to malted drink consumption (i.e. those who reported no malted drink consumption during the past 24 h may not imply that they did not consume it at all). Different nutritional assessment methods were used between age groups, though the study attempts to orient the respondent to recall/record the food accurately, nevertheless, under or over report may occurs and not reflecting actual food intake. In addition, parental reports of dietary intake may be subjected to errors if the children attended day care. Moreover, data on vitamin D intake should be interpreted cautiously. Values of vitamin D were obtained from nutrition information panels only which could lead to under-estimation. This is due to the unavailability of vitamin D content information in the Malaysian Food Composition database. Nevertheless, it highlights an important issue and concern about dietary vitamin D deficiency in our population. Ultimately, the cross-sectional nature of study design restricted our ability to establish causal inferences. It has been proposed that the relation between food intake and body weight may vary with different life stages due to alterations in physiological development, social and environmental factors [34]. Therefore, longitudinal studies are required to assess malted drink consumption pattern over time and health outcomes. Despite these limitations, the current study is important to provide valuable information to public health efforts targeting consumption of nutritious beverages.

## Conclusions

In conclusion, malted drink consumption is prevalent among Malaysian primary school children, particularly among boys, indigenous children and those who lived in the East Coast region of Malaysia. It was found that malted drink consumers had better micronutrient intakes and were more physically active than non-consumers of malted drinks. There was no observed association between malted drink consumption and weight status. These findings suggest that malted drinks are a micronutrient-rich beverage which are unlikely to promote excess energy intake and obesity risk, at the consumption pattern in the population assessed.

## Abbreviations

BAZ: BMI-for-age Z-scores
BMI: Body mass index
BMR: Basal metabolic rate
PAQ-C: Physical activity questionnaire for children
RNI: Recommended nutrient intakes
SMS: Short messaging system
UKMREC: Universiti Kebangsaan Malaysia Research Ethics Committee
