# Erratum to: ‘Characteristics associated with the consumption of malted drinks among Malaysian primary school children: findings from the MyBreakfast study’

**DOI:** 10.1186/s12889-016-2745-2

**Published:** 2016-02-16

**Authors:** Hamid Jan Jan Mohamed, S. L. Loy, Mohd Nasir Mohd Taib, Norimah A. Karim, S. Y. Tan, M. Appukutty, Nurliyana Abdul Razak, F. Thielecke, S. Hopkins, M. K. Ong, C. Ning, E. S. Tee

**Affiliations:** Nutrition Programme, School of Health Sciences, Universiti Sains Malaysia, 16150 Kubang Kerian, Kelantan Malaysia; KK Research Centre, KK Women’s and Children’s Hospital, Bukit Timah Road, Singapore, 229899 Singapore; Department of Nutrition and Dietetics, Faculty of Medicine and Health Sciences, Universiti Putra Malaysia, 43400 Serdang, Selangor Darul Ehsan Malaysia; School of Healthcare Sciences, Faculty of Health Sciences, Universiti Kebangsaan Malaysia, Jalan Raja Muda Abd Aziz, 50300 Kuala Lumpur, Malaysia; Department of Nutrition and Dietetics, School of Health Sciences, International Medical University, No.126, Jalan Jalil Perkasa 19, Bukit Jalil, 57000 Kuala Lumpur, Malaysia; Sports Science Programme, Faculty of Sports Science and Recreation, Universiti Teknologi MARA, 40450 Shah Alam, Selangor Darul Ehsan Malaysia; Cereal Partners Worldwide, Chemin du Viaduc 1. Prilly, Lausanne, 1008 Switzerland; Nestlé Research Center, Vers chez les Blanc, 1000 Lausanne, Switzerland; Nestlé R&D Center, 2655633, 29 Quality Road, 618802 Singapore, Singapore; Nutrition Society of Malaysia, c/o Division of Human Nutrition, Institute for Medical Research, Kuala Lumpur, Malaysia

Unfortunately, the original version of this article [1] contained an error. There was repetition of town name in affiliation number 8 and there was an error in the first author’s name. Both have been corrected below and also updated in the original article:

^8^Nestlé Research Center, Vers chez les Blanc, 1000 Lausanne, Switzerland

First name: Hamid Jan

Second name: Jan Mohamed
